# Membrane Retention of West Nile Virus NS5 Depends on NS1 or NS3 for Enzymatic Activity

**DOI:** 10.3390/v16081303

**Published:** 2024-08-16

**Authors:** Alanna C. Tseng, Vivek R. Nerurkar, Kabi R. Neupane, Helmut Kae, Pakieli H. Kaufusi

**Affiliations:** 1 Department of Tropical Medicine, Medical Microbiology and Pharmacology, John A. Burns School of Medicine, University of Hawaii at Manoa, Honolulu, HI 96813, USA; acytseng@hawaii.edu; 2 Molecular Biosciences and Bioengineering Graduate Program, College of Tropical Agriculture and Human Resources, University of Hawaii at Manoa, Honolulu, HI 96822, USA; 3 Pacific Center for Emerging Infectious Diseases Research, John A. Burns School of Medicine, University of Hawaii at Manoa, Honolulu, HI 96813, USA; 4 Division of Math and Sciences, Leeward Community College, Pearl City, HI 96782, USA; kabi@hawaii.edu (K.R.N.); helmut@hawaii.edu (H.K.)

**Keywords:** flavivirus, West Nile virus, endoplasmic reticulum, replication organelles, NS5, NS3, NS1, confocal microscopy, sucrose density gradient

## Abstract

West Nile virus (WNV) nonstructural protein 5 (NS5) possesses multiple enzymatic domains essential for viral RNA replication. During infection, NS5 predominantly localizes to unique replication organelles (ROs) at the rough endoplasmic reticulum (RER), known as vesicle packets (VPs) and convoluted membranes (CMs), with a portion of NS5 accumulating in the nucleus. NS5 is a soluble protein that must be in the VP, where its enzymatic activities are required for viral RNA synthesis. However, the mechanistic processes behind the recruitment of NS5 from the cytoplasm to the RER membrane remain unclear. Here, we utilize high-resolution confocal microscopy and sucrose density gradient ultracentrifugation to investigate whether the association of NS5 with other NS proteins contributes to its membrane recruitment and retention. We demonstrate that NS1 or NS3 partially influences the NS5 association with the membrane. We further demonstrate that processed NS5 is predominantly in the cytoplasm and nucleus, indicating that the processing of NS5 from the viral polyprotein does not contribute to its membrane localization. These observations suggest that other host or viral factors, such as the enwrapment of NS5 by the RO, may also be necessary for the complete membrane retention of NS5. Therefore, studies on the inhibitors that disrupt the membrane localization of WNV NS5 are warranted for antiviral drug development.

## 1. Introduction

West Nile virus (WNV), a mosquito-borne flavivirus, appeared in the US in 1999 and reemerged in 2012, resulting in over 58,900 clinical cases of WNV disease between 1999 and 2023 [1,2,3]. Although most human WNV infections are limited to mild febrile illness, some infected individuals, including immunocompromised patients, develop severe WNV neuroinvasive disease [4]. WNV infection in the brain can manifest as meningitis, encephalitis, or poliomyelitis-like syndrome and lead to long-term neurological complications [5,6], highlighting its potential as a continuing threat to public health.

WNV is an enveloped virus with a single-stranded, positive-sense RNA genome approximately 11 kb in length [7]. The viral genome is released into the cytoplasm of the infected cell, where it is directly translated at the rough endoplasmic reticulum (RER) into a large polyprotein. The polyprotein is cleaved into three structural proteins (capsid, precursor membrane, and envelope) and seven nonstructural proteins (NS1, NS2A, NS2B, NS3, NS4A, NS4B, and NS5) [8]. The structural proteins are the components of the virus particle and are involved in receptor binding and viral entry [9,10,11]. The NS proteins consist of membrane-associated proteins (NS1, NS2A, NS2B, NS4A, and NS4B) and soluble proteins (NS3 and NS5) [12,13,14]. The membrane-associated NS proteins not only support virus replication but are also involved in the remodeling of the RER membranes to generate replication organelles (ROs), which include convoluted membranes (CMs) and vesicle packets (VPs) [15,16,17,18,19]. The CM is considered the site for polyprotein processing, whereas the VP concentrates the components of the replication complex for viral RNA synthesis [20]. The soluble NS3 consists of a distinct protease domain for polyprotein processing as well as helicase and triphosphatase domains required for virus replication in the RO [21,22]. NS5, the largest and most conserved flaviviral protein, harbors the RNA methyltransferase (MTase) and RNA-dependent RNA polymerase (RdRP) domains essential for viral RNA synthesis [23,24].

NS5, a soluble protein, localizes at the RO during infection [25,26,27], and a portion of NS5 is found within the nucleus, where it has been demonstrated to antagonize host antiviral responses [28,29,30]. In the absence of the RO, NS5 localizes predominantly to the cytoplasm, and some translocates into the nucleus [31]. Therefore, the recruitment of NS5 protein to the RO and its retention in these compartments after it is processed from the polyprotein is critical for its enzymatic activities at the RER membrane [29]. The other NS proteins are in the RO [18,20], and their potential interaction with NS5 may modulate the RO retention of NS5. Although the mechanistic details behind these coordinated processes are largely unknown, studies have demonstrated that NS5 interacts directly with NS3 [32] at specific residues [14,33,34]. A mutation that disrupts the NS3–NS5 interaction reduces viral RNA replication and abolishes infectious virus production [35].

The role of NS3 and NS1 in the recruitment and retention of soluble NS5 at the RER membrane has yet to be explored. The presence of NS1 in the VP has been demonstrated to modulate NS5 function, but whether NS1 directly interacts with NS5 has not been established [36,37,38]. In this study, we examined whether the interactions of NS5 with other NS proteins (NS1, NS3, or NS4B) promoted its association with the membrane. We further tested whether the cleavage of NS5 from the viral polyprotein influenced the membrane association of NS5. Our results showed that the processing of WNV NS5 from the viral polyprotein did not influence its localization to the RER membrane. Once NS5 was cleaved, NS1 or NS3 partially contributed to its membrane association, suggesting that these viral proteins are involved in recruiting and retaining NS5 at the RO compartments.

## 2. Materials and Methods

### 2.1. Cell Culture and Virus

Low-passage human embryonic kidney 293T (HEK293T ATCC^®^ CRL-3216™) cells were propagated according to our previously described protocols [39,40]. For all the infection experiments, a stock of lineage I WNV strain NY99 (WNV_NY99_), originally isolated from a crow in New York and propagated in Vero cells, was used [41]. HEK293T cells infected at a multiplicity of infection (MOI) of 1 were either fixed for immunofluorescence or lysed for Western blot assays.

### 2.2. Plasmid Construction

As previously described, standard molecular biology techniques were used to clone the WNV nonstructural genes [40]. Briefly, the viral RNA extracted from the supernatant of WNV infected-Vero cells using the QIAamp Viral RNA mini kit (Cat#52904, Qiagen, Hilden, Germany) was used as a template to produce cDNA with the SuperScript IV first-strand synthesis kit (Cat#18091050, ThermoFisher Scientific, Waltham, MA, USA). The cDNA was used as the template for the polymerase chain reaction (PCR) amplification of the WNV NS genes. Both forward and reverse primers for each WNV NS gene were designed from a WNV_NY99_ reference sequence from NCBI (Table S1). The resulting PCR products were cloned into the pcDNA^TM^3.1/V5-His-TOPO (Cat#K480001, ThermoFisher Scientific, Waltham, MA, USA) or the pcDNA^TM^3.1/CT-GFP-TOPO (Cat#K482001, ThermoFisher Scientific, Waltham, MA, USA) vectors according to the manufacturer’s instructions. Plasmid DNA from *E. coli* cultures was purified using a plasmid Maxi Kit (Cat#12163, Qiagen, Hilden, Germany). The pcDNA^TM^3-IKKε-FLAG plasmid was constructed as previously described [42] and acquired through the Addgene repository (Cat#26201, Addgene, Watertown, MA, USA). Dr. Nihal Altan-Bonnett kindly provided the host marker plasmid Sec61β-RFP to detect the RER (National Institutes of Health, Bethesda, MD, USA).

### 2.3. Transient Transfections

HEK293T cells were transfected using the Polyfect transfection reagent (Cat#301105, Qiagen, Hilden, Germany) according to the manufacturer’s protocol. HEK293T cells were seeded in 24-well plates on 12 mm glass coverslips (Cat#CLS-1760-012, Chemglass, Vineland, NJ, USA) or 6-well plates and cultured in high-glucose Dulbecco’s modified Eagle medium (DMEM) (Cat#D5796, Millipore Sigma, Burlington, MA, USA) with 10% (*v*/*v*) fetal bovine serum (FBS) (Cat#MT35015CV, ThermoFisher Scientific, Waltham, MA, USA) for one day before transfection. A mixture containing 0.5–1.0 μg of total DNA plasmid per 3 × 10^5^ cells, Polyfect reagent, and growth medium without FBS was added to the cell culture. HEK293T cells were transfected with combinations of two, three, or four recombinant plasmids. Twenty-four hours after transfection, the cells were either fixed with 3.7% paraformaldehyde (PFA) in 1X PBS for immunofluorescence (IF) labeling or harvested for Western blot (WB).

### 2.4. Immunofluorescence Staining and Confocal Microscopy

The fixed HEK293T cells on coverslips were incubated with the appropriate primary and secondary antibodies (Table S2) to visualize both the viral and host proteins. The cells were incubated with primary antibodies (at indicated dilutions in Table S2) in 0.1% saponin (Cat#S4521, Millipore Sigma, Burlington, MA, USA), 2% bovine serum albumin (BSA) (Cat# 10735078001, Millipore Sigma, Burlington, MA, USA), and 1X PBS for 1 h. After three washes with 1X PBS, the cells were incubated with a secondary antibody (Table S2) in 2% BSA and 1X PBS for 45 min. All the incubations were performed at room temperature. The stained cells were washed three times with 1X PBS and mounted on slides using the Vectashield Mounting Medium with DAPI (Cat#H1200, Vector Laboratories, Burlingame, CA, USA). The cells were viewed and captured using the Olympus FV-1000 confocal laser scanning microscope (Olympus America Inc., Center Valley, PA, USA) with a 40× objective, and colocalization was confirmed with a 63× objective. The images were processed and merged with the Adobe Photoshop software, version 22.0.

### 2.5. Quantitation of Colocalization

The colocalization of immunofluorescent signals was analyzed using the Coloc 2 plug-in within the FIJI image analysis software, version 2.1.0/1.53c [42] (National Institutes of Health, Bethesda, MD, USA) as previously described [40]. The regions of interest were manually drawn around distinct cells to include the entire cytoplasm and exclude the nucleus. Pearson’s correlation coefficient (PCC) using the Costes’ automatic threshold [43] was calculated from 7 to 10 representative cells to measure the degree of colocalization between the viral proteins and host markers. The PCC values ranging from 0.5 to 1 indicated a high level of colocalization, 0.3 to 0.5 indicated moderate levels of correlation, and values less than 0.3 indicated low or negligible correlation.

### 2.6. Subcellular Fractionation

The infected or transfected HEK293T cells were fractionated as described previously [19] with slight modifications. Briefly, the cells from a confluent 100 mm plate were scraped and incubated in 500 µL of hypotonic buffer containing 1% proteinase inhibitor cocktail for 30 min on ice. The cells were homogenized and lysed by 20 passages through a 25-gauge needle (Cat#BD305127, BD Biosciences, San Jose, CA, USA), and the post-nuclear supernatant (PNS) was cleared by centrifugation for 5 min at 1000× *g* at 4 °C. Five hundred µL of PNS were layered onto a discontinuous density gradient consisting of seven sucrose layers of increasing molarity (0.2, 0.4, 0.6, 1.0, 1.4, 1.8, and 2.0 M, 400 µL each) and centrifuged for 2 h at 4 °C at 55,083× *g* in a Beckman SW55 rotor. Eleven fractions were collected from the top, and 20 µL of each fraction was used for the WB analysis.

### 2.7. Cell Lysis

At 48 h post-transfection, the cells seeded in 6-well plates were washed with ice-cold 1X PBS, detached using trypsin, and lysed for 1 h on ice using the M-PER^TM^ Mammalian Protein Extraction Reagent (Cat#78503, ThermoFisher Scientific, Waltham, MA, USA) containing 1% proteinase inhibitor cocktail (Cat#4693159001, Millipore Sigma, Burlington, MA, USA). The suspension was centrifuged at 15,000 rpm at 4 °C for 35 min, and the resulting pellet containing the nuclei and cellular debris was discarded. The protein concentration was determined using the Quick Start^TM^ Bradford Protein Assay kit (Cat#5000201, Bio-Rad, Hercules, CA, USA).

### 2.8. Western Blot

Approximately 20 μg of the total protein prepared with the 4X NuPAGE LDS sample buffer (Cat#NP0007, ThermoFisher Scientific, Waltham, MA, USA) was electrophoresed on 4–12% pre-cast NuPAGE gels (Cat#NP0322BOX, ThermoFisher Scientific, Waltham, MA, USA). The proteins were then transferred onto a nitrocellulose membrane (Cat#LC2006, ThermoFisher Scientific, Waltham, MA, USA) using the Trans-Blot Turbo Transfer System (Bio-Rad, Hercules, CA, USA) according to the manufacturer’s protocol. The membrane was blocked with the Li-Cor Blocking Buffer (Cat#927-60001, Li-Cor Biosciences, Lincoln, NE, USA) for 1 h at room temperature and incubated with primary antibodies (Table S2) at 4 °C overnight with gentle shaking. The membrane was washed five times with 1X TBS with 0.1% Tween^®^ 20 (TBST), followed by incubation with goat anti-rabbit IgG IRDye 800CW (Cat#926-32211) or goat anti-mouse IgG IRDye 680RD (Cat# 926-68170) secondary antibodies (Li-Cor Biosciences, Lincoln, NE, USA) for 45 min at room temperature. After five washes with TBST, the membranes were scanned using the Odyssey CLx Imaging system (Li-Cor Biosciences, Lincoln, NE, USA).

## 3. Results

### 3.1. NS5 Associates with Intracellular Membranes during Infection

**Figure 1:**
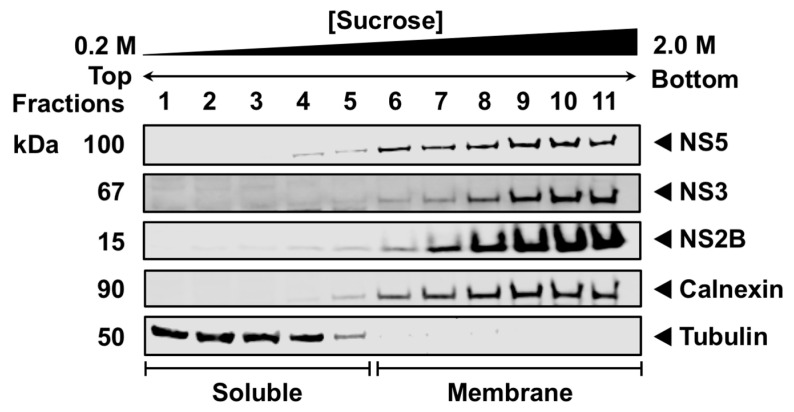
WNV NS5 is localized to the endoplasmic reticulum membranes during infection. HEK293T cells were infected with WNV_NY99_ at a MOI of 1 and harvested after 48 h. The cell fractions were separated using sucrose gradient ultracentrifugation and subjected to SDS-PAGE before immunoblotting. Viral proteins were detected using anti-WNV NS5, NS3, and NS2B antibodies. The lighter soluble fractions and heavier membrane fractions were visualized using antibodies against tubulin and calnexin, respectively.

Published data have demonstrated that NS5, a soluble protein, predominantly localizes in the RER where viral RNA replication occurs [18,20,44,45], but how NS5 is recruited and retained at the RER membrane during infection remains unknown. First, we examined the membrane association of WNV NS5 in the context of infection using subcellular fractionation and sucrose density gradient ultracentrifugation (SDGU). HEK293T cells were infected with WNV_NY99_ for 48 h, and the cell organelles were separated using a hypotonic buffer. The nuclei and cellular debris were discarded, and the cleared organelle suspension was layered onto a discontinuous sucrose gradient. Western blot analysis of the fractions collected from the top of the gradient indicated a clear separation between the soluble (fractions 1–5) and membrane fractions (fractions 6–11) using antibodies against tubulin-β and calnexin, respectively (Figure 1). Tubulin-β, a major soluble component of the microtubules that form the cytoskeleton [46], was used to label the lighter fractions, whereas calnexin, an integral membrane protein in the ER [47], was utilized to identify the heavier RER membrane-containing fractions. Because NS3 and NS2B have been demonstrated to localize to the RO at the RER during infection, NS3 and NS2B were used as positive controls for the membrane fractions in the infected cells [40]. As expected, NS3 and NS2B were entirely in the membrane fractions (fractions 6–11). Similarly, NS5 was highly enriched in the membrane (fractions 6–11) but not in the soluble fractions (fractions 1–5) (Figure 1), confirming that NS5 was primarily confined to the intracellular membranes during infection. The NS3, NS2B, calnexin, and tubulin panels were derived from our previous publication [40].

### 3.2. NS5 Is Distributed Throughout the Cytoplasm and Nucleus in Transfected Cells

**Figure 2:**
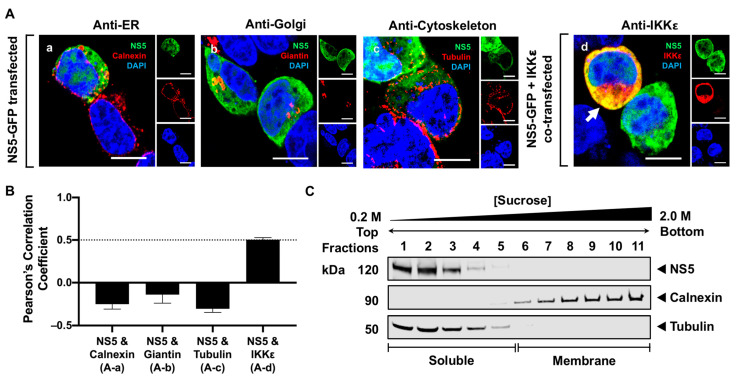
WNV is localized to the cytoplasm and nucleus in the transfected cells. (**A**) (**a**–**c**) NS5-GFP-transfected or (**d**) NS5-GFP and IKKε-FLAG co-transfected HEK293T cells were fixed at 24 h post-transfection and immunostained with antibodies against (**a**) calnexin (red), (**b**) giantin (red), (**c**) tubulin (red), or (**d**) IKKε (red). Antibodies that detect the host markers and the viral protein’s GFP tag (green) are listed in the top right corner of each panel. Nuclear DNA (blue) was stained with 4,6-diamidino-2-phenylindole (DAPI). The confocal microscopy images were of optical slice thickness ~1 μm. Scale bar, 10 μM. The main image depicts the merged image from the three separate channels shown on the side. The white arrow indicates colocalization between the green and red channels. (**B**) The Pearson’s correlation coefficient (PCC) analysis of the colocalization between NS5 and cellular markers. PCC values at 0.5 (dotted line) indicate a high level of colocalization. Letters in parenthesis correspond to its representative confocal image in Figure 2A. Error bars indicate the mean ± standard error of the mean (SEM); *n* = 7–10 cells per group. (**C**) HEK293T cells were transfected with NS5-GFP for 48 h and subjected to subcellular fractionation followed by sucrose gradient centrifugation. The cell fractions were analyzed by Western blot using rabbit anti-GFP antibody to detect NS5. The membrane and soluble fractions were detected using antibodies against calnexin and tubulin, respectively.

Due to the soluble nature of NS5, we next examined whether NS5, in the absence of other viral proteins, is localized to the cytoplasm. We co-stained a GFP-fused NS5 with different cellular markers 24 h after expression and visualized the localization of NS5 using high-resolution confocal microscopy (HRCM). The RER, Golgi, and cytoskeleton were stained using antibodies against calnexin, giantin, and tubulin, respectively. The majority of NS5 appeared to distribute throughout the cytoplasm and a fraction in the nucleus, but none was observed in the RER, Golgi, or cytoskeleton (Figure 2(Aa–Ac)). To verify that NS5 was scattered in the cytoplasm, NS5 and IKKε were co-expressed in HEK293T cells for 24 h and detected using antibodies against GFP and IKKε, respectively. IKKε is a soluble host protein kinase known to localize in the cytoplasm [42]. NS5 colocalized with the IKKε protein, confirming that NS5 is a soluble protein diffusely distributed in the cytoplasm (Figure 2(Ad)). We quantitatively assessed the colocalization between NS5 and each host marker by calculating Pearson’s correlation coefficient (PCC). Consistent with our visual observations, only the PCC for NS5 and IKKε was high, with a mean value of 0.5 ± 0.2 (Figure 2B). We further used SDGU to separate the cytoplasm from the membrane fractions. Because tubulin and IKKε are predominantly found in the cytoplasm, either tubulin or IKKε can be used to define the soluble fractions. Western blot analysis of the fractionated gradients confirmed that NS5, when individually expressed, was only detected in the soluble fractions (fractions 1–4) (Figure 2C). Together, the results from HRCM and SDGU demonstrate that NS5, in the absence of other viral proteins, is diffusely distributed in the cytoplasm.

### 3.3. Processing of NS5 from the Viral Polyprotein Does Not Contribute to Its Membrane Localization

**Figure 3:**
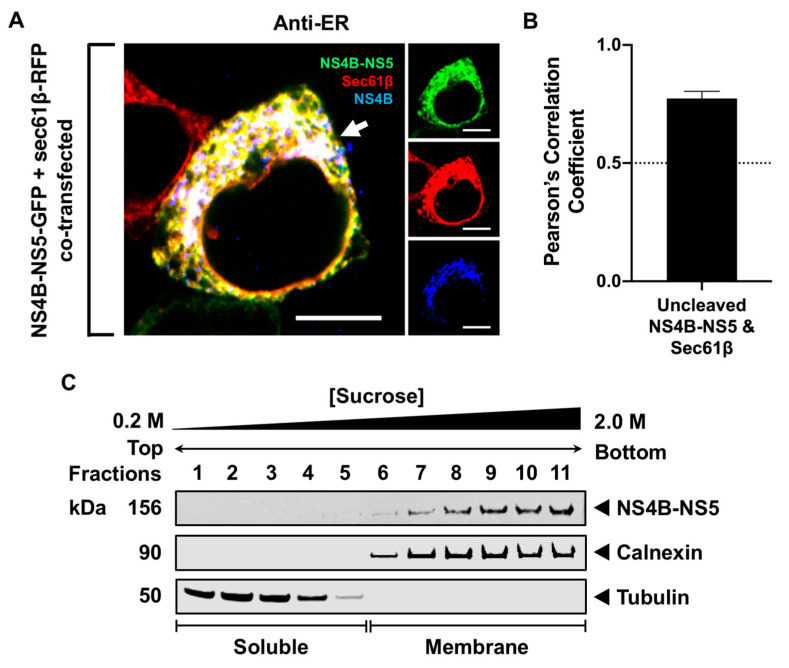
WNV NS5 is associated with the RER when it is tethered to NS4B. (**A**) The NS4B-NS5-GFP (green) and sec61β-RFP (red) co-transfected HEK293T cells were fixed 24 h after transfection and immunolabeled with anti-JEV NS4B (blue). Optical slice thickness ~1 μm. Scale bar, 10 μM. The white arrow indicates colocalization between all three channels. The image is representative of three independent transfection experiments. (**B**) Pearson’s correlation coefficient between NS4B-NS5 and sec61β (RER marker). PCC values greater than 0.5 (dotted line) indicate a high level of colocalization. Error bars indicate mean ± SEM; *n* = 10 cells. (**C**) The cells transiently expressing NS4B-NS5-GFP were harvested after 48 h. The cell fractions separated using SDGU were immunoblotted with antibodies against GFP, calnexin, and tubulin.

Since NS5 is predominantly in the cytoplasm during transfection but observed exclusively in the membrane during infection, we examined whether the localization of NS5 to the membrane is influenced by NS4B, given that NS4B is a membrane protein preceding NS5. We constructed a plasmid containing NS4B-NS5 with a GFP fusion to examine its intracellular localization (Figure 3). NS4B-NS5-GFP and Sec61β-RFP were co-expressed in HEK293T cells. Sec61β, a subunit of the protein translocation apparatus in the ER membrane, is another commonly used RER marker [48]. The colocalization between NS4B-NS5-GFP and Sec61β-RFP was visualized directly using HRCM and quantitated using the PCC analysis. At 24 h, the fixed cells were stained with an NS4B antibody to visually confirm the RER localization of NS4B-NS5. The full-length, uncleaved NS4B-NS5 GFP-fused polyprotein colocalized with Sec61β (PCC = 0.77 ± 0.03), confirming that NS4B-NS5 remained in the RER (Figure 3A,B). The association of NS4B-NS5-GFP with the membrane was also verified using SDGU. Western blot analysis of the fractions collected from the NS4B-NS5-GFP-transfected HEK293T cells showed that NS4B-NS5 was only in the calnexin-enriched membrane fractions (fractions 7–11) (Figure 3C). These observations indicate that NS4B-NS5 with the GFP fusion still associates with the RER, similar to its wild-type counterpart in infected cells.

**Figure 4:**
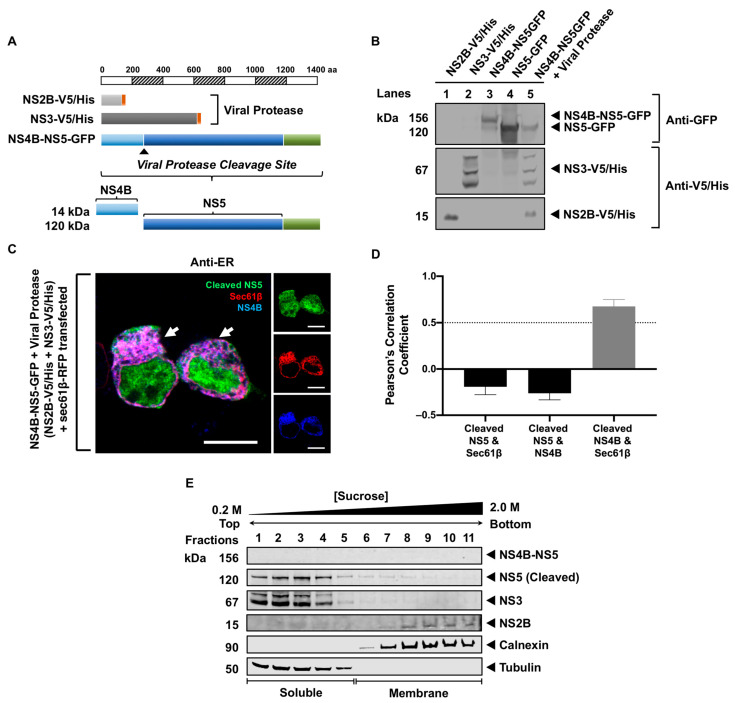
Processing of WNV NS5 from the NS4B-NS5 polyprotein does not influence its RER localization. (**A**) The schematic representation of the V5/His (red box) or GFP-tagged (green box) WNV NS constructs used in this assay. Constructs are drawn to scale according to the number of amino acid residues. (**B**) HEK293T cells were transfected with various WNV NS constructs (listed at the top of the panel), and lysates were harvested 48 h post-transfection. The cleavage of NS4B-NS5-GFP by the viral protease (NS3-V5/His with NS2B-V5/His provided in trans) in lane 5 was assessed by Western blot. (**C**) HEK293T cells were transfected with the NS4B-NS5-GFP (green), NS2B-V5, NS3-V5, and sec61β-RFP (red) plasmids, and the quadruple-transfected cells were processed for immunofluorescence 24 h after transfection. Optical slice thickness ~1 μm. Scale bar, 10 μM. White arrows indicate colocalization between sec61β-RFP and NS4B (blue). (**D**) Colocalization analysis between the cleaved NS5 protein, sec61β, and NS4B. Error bars indicate mean ± SEM; *n* = 8 cells. PCC values above 0.5 (dotted line) indicate higher levels of colocalization. (**E**) Cell fractions from cells expressing NS4B-NS5-GFP, NS2B-V5, NS3-V5, and sec61β-RFP 48 h post-transfection were analyzed by Western blot. The cleaved NS5 was detected using an anti-GFP antibody and the NS2B and NS3 proteins were visualized using anti-V5/His antibodies. The membrane and soluble fractions were distinguished using anti-calnexin and anti-tubulin antibodies, respectively.

We next examined whether NS5 remained in the RER after cleavage from the viral polyprotein and characterized the processing of NS4B-NS5-GFP with NS2B-V5/His and NS3-V5/His supplied in trans in HEK293T cells (Figure 4A). At 48 h after transfection, Western blot analysis was performed on the cell lysates using antibodies against GFP and V5/His epitopes. NS4B-NS5-GFP (~156 kDa) was processed by the viral protease consisting of NS2B-V5/His (~15 kDa) and NS3-V5/His (~67 kDa), releasing NS5-GFP (~120 kDa) (Figure 4B, lane 5). The single transfections of NS2B-V5/His, NS3-V5/His, NS4B-NS5-GFP, and NS5-GFP served as controls (Figure 4B, lanes 1–4). Next, we examined whether the cleaved NS5-GFP localized to the RER membrane using HRCM and PCC analysis. We performed a quadruple transfection of NS4B-NS5-GFP, NS2B-V5/His, NS3-V5/His, and Sec61β-RFP in HEK293T cells. Using an antibody against NS4B (Table S2), we demonstrated that NS4B, as expected, colocalized with the RER marker, sec61β (PCC = 0.67 ± 0.08) (Figure 4C,D). In contrast, cleaved NS5-GFP was distributed throughout the cytoplasm and the nucleus and did not appear to colocalize with the RER (Figure 4C). The association of the cleaved NS5-GFP with the cytoplasm was also confirmed using SDGU (Figure 4E). The soluble and membrane fractions were visualized using tubulin and calnexin antibodies, respectively. NS4B-NS5-GFP was not detected, indicating that the viral protease completely processed the polyprotein. NS5-GFP was detected in the soluble fractions together with NS3-V5 (fractions 1–5), while NS2B-V5 was in the membrane fractions (fractions 7–11) (Figure 4E). These observations imply that the processing of NS5 from the viral polyprotein during infection was not involved in its membrane localization.

### 3.4. NS5 Partially Associates with the RER Membrane in the Presence of NS1 or NS3 Proteins

**Figure 5:**
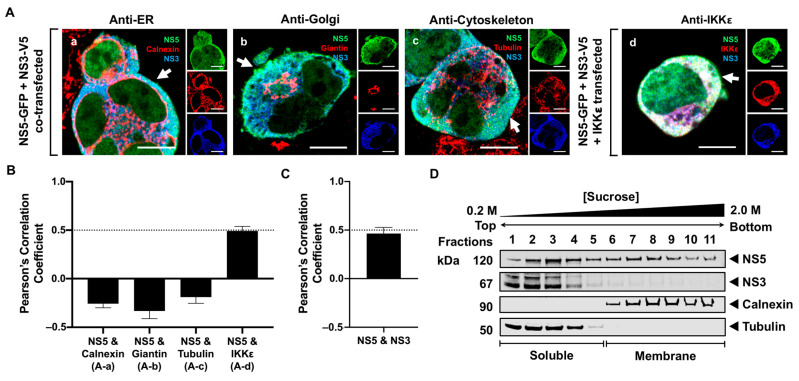
WNV NS5 predominantly localizes in the cytoplasm and partially localized in the membrane fractions when NS3 is present. (**A**) HEK293T cells were co-transfected with (**a**–**c**) the NS5-GFP (green) and NS3-V5/His (blue) plasmids or (**d**) triple-transfected with the NS5-GFP, NS3-V5/His and IKKε-FLAG plasmids. The cells were processed for immunofluorescence 24 h after transfection using antibodies against (**a**) calnexin (red), (**b**) giantin (red), (**c**) tubulin (red), or (**d**) IKKε (red). White arrows depict colocalization between (**a**–**c**) the green and blue channels or (**d**) all three channels. Scale bar, 10 μM. (**B**,**C**) Pearson’s correlation coefficient to measure colocalization was determined for each indicated pair of proteins. PCC values at 0.5 (dotted line) indicate a high level of colocalization. Parenthesized letters link each PCC value to its corresponding image in Figure 5A. The PCC for NS5 and NS3 was calculated using the co-transfected (**a**–**c**) and triple-transfected (**d**) cells. Error bars indicate mean ± SEM; *n* = 7–10 cells per group. (**D**) Subcellular fractions from cells expressing NS5-GFP with NS3-V5/His provided in trans 48 h post-transfection were analyzed using antibodies against the fused tag (GFP or V5/His) to detect the viral proteins. Calnexin and tubulin indicate the membrane and soluble fractions, respectively.

Since NS5 is released to the cytoplasm after it is processed from the viral polyprotein, we next examined whether other NS proteins influenced its retention at the RER membrane. Previously published data have demonstrated that NS3 directly interacts with NS5 [35,36], suggesting that NS3 may influence the membrane localization of NS5. To determine whether NS3 was involved in the retention of NS5 to the RER, we constructed a plasmid containing NS3 with a V5/His tag fusion. NS5-GFP was co-expressed with NS3-V5/His in HEK293T cells and fixed 24 h after transfection (Figure 5). The cells were stained with antibodies against V5 to detect NS3 or against calnexin, giantin, and tubulin to detect the RER, Golgi, and cytoskeleton, respectively. Triple-channel confocal imaging and quantitative PCC analysis revealed that NS5 remained in the cytoplasm, and none was observed in the RER, Golgi, or cytoskeleton in the presence of NS3 (Figure 5(Aa–Ac)). To further confirm the NS5 localization in the cytoplasm, HEK293T cells were triple-transfected with the NS5-GFP, IKKε, and NS3-V5/His plasmids for 24 h and detected using the anti-IKKε and V5/His antibodies. NS5 colocalized with IKKε (PCC = 0.49 ± 0.05; Figure 5B) and NS3 (PCC = 0.47 ± 0.09; Figure 5C), indicating that NS5 and NS3 both remained in the cytoplasm. Subcellular fractionation and SDGU were subsequently performed on the cells co-expressing NS5 and NS3. Western blot analysis of the subcellular fractions demonstrated that NS3 remained in the soluble fractions (fractions 1–5), which was consistent with the immunofluorescence data. However, NS5 was distributed in both soluble (fractions 1–5) and membrane fractions (fractions 6–10), indicating that NS3 may contribute partly to the association of NS5 with the intracellular membranes (Figure 5D). The conflicting results from the IF and SDGU data suggest that our biochemical assay may be more suitable for detecting the association of NS5 with the intracellular membrane in the presence of NS3. These observations also indicate that NS3 may indirectly influence host factors or other viral proteins that may also be involved in the membrane association of NS5.

**Figure 6:**
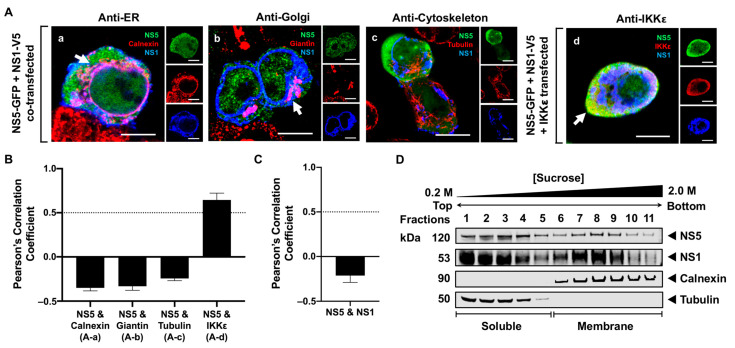
WNV NS5 is distributed throughout the cytoplasm and nucleus, and is slightly enriched in the membrane fractions when NS1 is present. (**A**) HEK293T cells were co-transfected with (**a**–**c**) the NS5-GFP (green) and NS1-V5/His (blue) plasmids or (**d**) triple-transfected with the NS5-GFP, NS1-V5/His and IKKε-FLAG plasmids. The cells fixed 24 h after transfection were immunostained with antibodies against (**a**) calnexin (red), (**b**) giantin (red), (**c**) tubulin (red), and (**d**) IKKε (red). White arrows depict colocalization between (**a**,**b**) the red and blue channels or (**d**) between the red and green channels. Scale bar, 10 μM. Pearson’s correlation coefficient was calculated for (**B**) NS5 and the host markers in the presence of NS1 and for (**C**) NS5 and NS1. PCC values greater than 0.5 (dotted line) indicate high levels of colocalization. Letters in parenthesis correlate each PCC value to its representative image in (**A**). Error bars indicate mean ± SEM; *n* = 7–10 cells per group. Error bars indicate mean ± SEM; n = 10 cells. (**D**) The cellular fractions from HEK293T cells co-expressing NS5-GFP with NS1-V5/His were analyzed using antibodies against the fused tag (GFP or V5/His) to detect the viral proteins, and against calnexin or tubulin to visualize the membrane and soluble fractions, respectively.

Previous studies have also demonstrated that NS1 is an essential cofactor for viral RNA synthesis [49], suggesting that NS1 can indirectly modulate NS5 function. Additionally, NS1 is localized to various sites within infected cells, including the RER, Golgi, and plasma membrane, and it is also secreted into the extracellular space [50]. Therefore, we explored whether NS1 plays a role in the membrane localization of NS5 by constructing and expressing an NS1-V5/His plasmid with NS5-GFP in HEK293T cells. At 24 h after transfection, the cells were fixed and immunostained with anti-V5, anti-calnexin, anti-giantin, and anti-tubulin antibodies to detect NS1, RER, Golgi, and cytoskeleton, respectively (Figure 6A). The colocalization between NS5 and the host markers was also quantitatively verified using PCC (Figure 6B). NS5 was observed to remain in the cytoplasm, and none was observed in the RER, Golgi, or cytoskeleton, despite the visual observation of NS1 at the RER and Golgi apparatus (Figure 6(Aa–Ac)). Additionally, the triple-channel imaging of the cells transfected with NS5-GFP, IKKε, and NS1-V5/His plasmids showed that NS5 colocalized with IKKε (PCC = 0.65 ± 0.08), but not with NS1 (Figure 6(Ad),C). Surprisingly, the biochemical analysis of the NS5-GFP and NS1-V5/His co-transfected cells using SDGU detected NS5 in both the soluble (fractions 1–5) and membrane fractions (fractions 6–11) (Figure 6D). Within the membrane fractions, NS5 appeared to be concentrated in fractions 7–9, coinciding with increased amounts of NS1 (Figure 6D). The association of NS5 with the membrane fractions in the presence of NS1 using SDGU indicates that NS1 may indirectly influence the association of NS5 with the cellular membranes. Taken altogether, the presence of NS1 or NS3 appeared to indirectly contribute to the partial membrane association of NS5.

## 4. Discussion

NS5 is the largest and most conserved viral NS protein, consisting of an N-terminal methyltransferase (MTase), guanylyltransferase (GTase), and a C-terminal RNA-dependent RNA polymerase (RdRP) [24]. The MTase and GTase are essential in forming the viral RNA cap, and the RdRP is vital for synthesizing new viral RNA during RNA replication. These enzymatic activities occur within the membrane structures at the RER, known as replication organelles (ROs), which are believed to be virally induced. Although the biogenesis and composition of the RO have not been completely characterized, two compartments observed within these membrane structures are vesicle packets (VPs) and convoluted membranes (CMs) [18,44]. Viral RNA synthesis occurs in the VP, where NS5 is expected to be localized [15,16]. Unlike the VP structures, the CM contains abundant viral NS proteins with a morphology that resembles smooth ER and lacks ribosomes [44], arguing that it may be a structure enriched with lipids that may promote the recruitment and stabilization of the viral RdRP from the cytoplasm [17]. Consistent with these studies, flavivirus NS5 is a cytoplasmic protein that is also present in the CM structures during infection [25,44]. How NS5 is recruited from the cytoplasm and retained at the RO to exert its enzymatic functions has not yet been explored. In this report, we apply HEK293T cell-transfection-based studies, high-resolution confocal microscopy (HRCM), and biochemical assays to investigate the role of viral NS proteins and polyprotein processing in understanding the recruitment of NS5 to the membrane.

Using sucrose density gradient ultracentrifugation (SDGU), we observed that NS5 is exclusively associated with the membrane but not the soluble fractions during infection (Figure 1), indicating that NS5 is recruited and well-retained in the virus-induced membrane structures, and some viral or host factors, or both, are involved in this process. Additionally, the absence of NS5 in the cytoplasm suggests that only a small proportion of NS5 possibly escapes the membrane during infection. This claim aligns with earlier studies using confocal microscopy on WNV Kunjin strain (KUN)-infected cells, showing that only a small fraction of NS5 was observed in the cytoplasm [25,51]. To further characterize NS5 in more detail, we expressed NS5 without other viral proteins and observed its localization to various sites within the cell using HRCM. NS5 is exclusively localized in the cytoplasm and nucleus, and none was detected in the membrane fractions (Figure 2). These data are consistent with a study showing NS5 nuclear localization in KUN-infected cells [31]. These observations indicate that additional viral factors present during infection are critically needed for NS5 membrane association.

The proper processing of the flavivirus polypeptide is critical for the function of the mature viral proteins [52,53]. We have previously demonstrated that sequential NS4A-NS4B processing significantly enhances immunomediator induction in transfected monocytes [54]. This observation led us to examine whether the processing of NS5 from the NS4B-NS5 polypeptide is involved in its membrane association. The co-expression of NS4B-NS5 polypeptide with the viral protease showed proper processing, releasing NS5. However, the cleaved NS5 was not observed in the RER membrane, but predominantly in the cytoplasm, with a small fraction in the nucleus. These results imply that the processed NS5 preferentially localizes to the cytoplasm and nucleus. The presence of the cleaved NS4B, a transmembrane protein [13], was also not involved in NS5 membrane localization (Figure 4). Interestingly, NS5 was associated with the membrane only when it was still attached to NS4B (Figure 3), suggesting that the processing of NS4B-NS5 polypeptide has to occur in conjunction with other viral factors or the formation of the replication complexes to trap and retain the cleaved NS5 at the membrane.

Our previous data revealed that NS3, a soluble protein, only associates with the RER in the presence of its NS2B cofactor [40]. Published data have shown that NS3 modulates NS5 function [35,36], implying that the presence of NS2B and NS3 may influence the NS5 association with the virus-induced membrane structures. However, our data on NS4B-NS5 processing showed that in the presence of NS2B and NS3, cleaved NS5 is predominantly in the cytoplasm (Figure 4). When NS3 engages with its NS2B cofactor for proteolytic activity [55], NS3 presumably becomes inaccessible to interact with NS5 and/or other viral proteins. However, when NS3 and NS5 are co-expressed, a portion of NS5 is found in the membrane fractions. HRCM confirmed that NS3 and NS5 colocalized with each other, but not in the RER (Figure 5). These observations indicate that in the absence of NS2B, NS3 may more freely associate peripherally with NS5 to allow the helicase domain of NS3 to interact with the NS5 polymerase for viral RNA synthesis [35].

The partial membrane association of NS5 in the presence of NS3 suggests that other viral proteins may also contribute to the localization of NS5 to the membrane. NS1, a primarily RER-resident protein, assumes multiple structural conformations that facilitate its localization to different cellular compartments and plays an essential cofactor role in NS5-mediated viral RNA synthesis [36,49]. Given these multifaceted characteristics of NS1, it is possible that NS1 may influence the membrane localization of NS5. As expected, NS1 was observed in the RER and Golgi using HRCM and was equally distributed in both the soluble and membrane fractions (Figure 6). Here, we show using HRCM that NS5 was predominantly observed in the cytoplasm and did not colocalize with NS1 or the RER. In contrast, our biochemical assay demonstrated that NS5 was in both the membrane and soluble fractions in the presence of NS1 (Figure 6), which provided evidence for NS1 in promoting the membrane localization of NS5. The discrepancy between the HRCM and biochemical data in our study highlights the constraints associated with the HRCM assay, which is limited by the use of highly specific host markers. It is clear from the biochemical data that NS5 is associated with intracellular membranes in the presence of NS1 or NS3 (Figure 5D and Figure 6D), but the composition of these NS5-associated membranes in our in vitro system may involve membrane proteins other than the conventional RER marker used in this study. The NS5-associated membranes here may more closely reflect the atypical composition of proteins and lipids observed in newly formed membrane structures in flavivirus-infected cells [17]. Studies examining the cellular components of flavivirus-modified membranes revealed that some membrane structures resembled smooth ER membranes [44] or were highly enriched with lipids [17] to form a microenvironment conducive to the stabilization of the viral RdRP for virus replication.

Taken altogether, NS5 partially depends on both NS1 and NS3 and their potential interactions with unknown host proteins to associate with the membrane. Flaviviruses have been shown to exploit the host lipid kinase PI4KIIIβ in order to generate PI4P lipid-enriched membranes to which the soluble viral RdRp can bind to initiate viral RNA synthesis [17]. Other studies have also demonstrated that flavivirus NS3 and NS5 interact with heterogeneous nuclear ribonucleoprotein F (HNRPF) and Golgi-associated PDZ and coiled-coil motif-containing protein (GOPC) to promote the formation of the viral replication complexes [56]. Furthermore, in accordance with the published work on the NS1 interactome [57], NS1 may also indirectly coordinate the recruitment of NS5 to the membrane through a concerted web of interactions with membrane-associated host factors. Our future studies will focus on dissecting the role of PI4KIIIβ and other lipid-related host components in the formation of NS5-associated membranes and characterizing the function of host factors such as HNRPF and GOPC in the recruitment of NS5 to the membrane.

## 5. Conclusions

To reconcile our results with the published data, we conclude that recruiting NS5 to the membrane, leading to its association with the RO, is an intricate process. The processing of NS5 from the WNV polyprotein is not involved, but the formation of the RO compartments may be a prerequisite for NS5 to be trapped and retained at the membrane once it is cleaved from NS4B. It is possible that NS5 processing coincides with RO formation, where multiple viral and host proteins may assist in the association of NS5 with the RER during infection. We show that NS1 and NS3, but not NS2B or NS4B, participate in the membrane localization of NS5, where its enzymatic functions are required. Additional studies using multiple membrane and lipid markers are needed to dissect the composition of the membranes involved in RO biogenesis. Further studies that elucidate the virus–host factors involved in the multifaceted process behind NS5 recruitment and retention in the membrane compartments are warranted to reveal effective targets for WNV antiviral drugs.

## Data Availability

The original contributions presented in the study are included in the article and Supplementary Materials. Further inquiries can be directed to the corresponding authors.

## Supplementary Materials

The following supporting information can be downloaded at https://www.mdpi.com/article/10.3390/v16081303/s1, Supplementary Table S1. Primer sequences used for the construction of WNV nonstructural (NS) gene plasmids. Supplementary Table S2. Antibodies and dilutions used for immunofluorescence and western blot staining.
