# Supplementary material for: Membrane Retention of West Nile Virus NS5 Depends on NS1 or NS3 for Enzymatic Activity
Source: Viruses. 2024 Aug 16;16(8):1303. doi: 10.3390/v16081303 (PMC11360346; doi:10.3390/v16081303)
Supplement: Supplementary file 1 [file viruses-16-01303-s001.zip › viruses-3129301-supplementary.pdf]

SUPPLEMENTARY FIGURES

Supplementary Table S1. Primer sequences used for the construction of WNV nonstructural (NS) gene plasmids.

WNV gene constructs	Primer	Nucleotide Position*	Primer Sequence (5′ → 3′)
**NS1**	forward	2398 – 2418	accaccatg[a]ATAGGTCCATAGCTCTCACG
	reverse	3502 – 3525	AGCATTCACTTGTGACTGCACGAG
**NS2B**	forward	4219 – 4234	accaccatg[a]GGATGGCCCGCAACTG
	reverse	4686 – 4611	TCTCTTTGTGTATTGGAGAGTTATCC
**NS3**	forward	4612 – 4631	accaccatg[a]GGAGGCGTGTTGTGGGACAC
	reverse	6444 – 6468	GACGTTTTCCCGAGGCGAAGTCCTTG
**NS4B-NS5**	forward[b]	6865 – 6882	accaccatg[a]*gct*[c]CTAGCCGTGTTCCTGATT
	reverse	10370 – 10395	GCAGTACTGTGTCCTCAACCAAAGTTG
**NS5**	forward	7681 – 7698	accaccatg[a]GTGGGGCAAAAGGACGC
	reverse	10370 – 10395	GCAGTACTGTGTCCTCAACCAAAGTTG

[a]Kozak sequence is lowercase, and the viral sequences are in uppercase. [b]NS4B forward primer includes 17-aa signal sequence at the -COOH end of NS4A. [c]Codon filling nucleotides are lowercase and italicized. *GenBank Accession No. DQ211652.

Supplementary Table S2. Antibodies and dilutions used for immunofluorescence and western blot staining.

Protein	Primary Ab	Catalog Number	IF	WB	Secondary Antibody	Catalog Number	IF	WB
			Dilutions				Dilutions	
**WNV NS5**	rabbit pAb	GTX131961[a]		1:3,000	anti-rabbit IRDye 800CW	926-32211[b]		1:10,000
**WNV NS3**	rabbit pAb	GTX131955[a]		1:3,000	anti-rabbit IRDye 800CW	926-32211[b]		1:10,000
**WNV NS2B**	rabbit pAb	GTX132060[a]		1:3,000	anti-rabbit IRDye 800CW	926-32211[b]		1:10,000
**JEV NS4B**	rabbit pAb	GTX125865[a]		1:4,000	anti-rabbit IRDye 800CW	926-32211[b]		1:10,000
			1:100		anti-rabbit IgG Pacific Blue	P10994[b]	1:500	
**Calnexin**	rabbit pAb	C4731[c]	1:100		anti-rabbit IgG AF 555	A21428[d]	1:1,000	
				1:2,000	anti-rabbit IRDye 800CW	926-32211[b]		1:10,000
**Giantin**	rabbit pAb	ab80864[e]	1:100		anti-rabbit IgG AF 555	A21428[d]	1:1,000	
**Tubulin β**	rabbit pAb	RB9249[d]	1:100		anti-rabbit IgG AF 555	A21428[d]	1:1,000	
	mouse mAb	sc-5274[f]		1:1,000	anti-mouse IRDye 680RD	926-68170[b]		1:10,000
**IκB kinase subunit ε (IKKε)**	rabbit mAb	2905S[g]	1:100		anti-rabbit IgG AF 555	A21428[d]	1:1,000	
**Green fluorescent protein (GFP)**	rabbit pAb	G10362[d]	1:100		anti-rabbit IgG AF 488	A11008[d]	1:1,000	
				1:4,000	anti-rabbit IRDye 800CW	926-32211[b]		1:10,000
**V5/His epitope**	mouse mAb	R960-25[c]	1:100		anti-mouse IgG Pacific Blue	P31582[b]	1:500	
				1:2,000	anti-mouse IRDye 680RD	926-68170[b]		1:10,000

[a]Genetex, Irvine, CA, USA; [b]Li-Cor Biosciences, Lincoln, NE, USA; [c]Millipore Sigma, Burlington, MA, USA; [d]ThermoFisher Scientific, Waltham, MA, USA; [e]Abcam, Cambridge, MA, USA; [f]Santa Cruz Biotechnology, Dallas, TX, USA; [g]Cell Signaling Technology, Danvers, MA, USA; pAB, polyclonal antibody; mAb, monoclonal antibody; IF, immunofluorescence; WB, Western blot; AF, Alexa Fluor.
